# CFTR Modulator Therapy and Aerobic Fitness Changes in Aerobic Fitness Following Initiation of CFTR Modulator Therapy: A Systematic Review and Meta‐Analysis

**DOI:** 10.1002/ppul.71826

**Published:** 2026-09-03

**Authors:** Ingrid Silveira de Almeida, Fernanda Maria Vendrusculo, Mariana Severo da Costa, Márcio Vinícius Fagundes Donadio

**Affiliations:** ^1^ Laboratory of Physical Activity in Pediatrics, Centro Infant Pontifícia Universidade Católica do Rio Grande do Sul (PUCRS) Porto Alegre Rio Grande do Sul Brazil; ^2^ Department of Physiotherapy, Faculty of Medicine and Health Sciences Universitat Internacional de Catalunya (UIC) Barcelona Spain

**Keywords:** aerobic fitness, CFTR modulator therapy, cardiopulmonary exercise testing, cystic fibrosis, oxygen consumption

## Abstract

**Introduction:**

Cystic fibrosis transmembrane conductance regulator (CFTR) modulator therapy has led to relevant clinical advances in people with cystic fibrosis (pwCF). However, its effects on aerobic fitness are unclear.

**Objective:**

To evaluate the effects of CFTR modulator therapy on aerobic fitness in pwCF through a systematic review and meta‐analysis.

**Methods:**

This systematic review (CRD420251066602) was conducted by searching major databases without date or language restrictions. Analytical studies evaluating cardiopulmonary exercise testing outcomes before and after CFTR modulator therapy were included. The primary outcome was peak oxygen uptake (peak VO_2_). Secondary outcomes included peak VO_2_ (% predicted), VO_2_ at the first ventilatory threshold (VT_1_), breathing reserve, workload, forced expiratory volume in one second (FEV_1_), and body mass index. Meta‐analyses were performed using a random‐effects model with heterogeneity assessed by the *I* ^2^ statistic, and sensitivity analyses conducted.

**Results:**

Thirteen studies (*n* = 578 participants) were included, of which 11 were eligible for meta‐analysis. The meta‐analysis did not identify a significant improvement in peak VO_2_ (mL·kg^−1^·min^−1^) after CFTR modulator use (MD = −0.22; 95% CI: −2.10 to 1.65; *p* = 0.82). For submaximal outcomes, after sensitivity analysis, VO_2_ at VT_1_ was significantly lower in the post‐intervention period (MD = −1.85; 95% CI: −2.61 to −1.10; *p* < 0.00001). Breathing reserve increased significantly (MD = 7.94; 95% CI: 2.15 to 13.73; *p* = 0.007), and workload also increased significantly (MD = 15.39; 95% CI: 2.20 to 28.58; *p* = 0.02).

**Conclusion:**

CFTR modulator therapy is not consistently associated with improvements in aerobic fitness, as assessed by peak VO_2_, although it appears to variably influence outcomes related to ventilatory response and exercise performance.

## 1 Introduction

Cystic fibrosis (CF) is a rare, autosomal recessive genetic disease caused by mutations in the gene encoding the cystic fibrosis transmembrane conductance regulator (CFTR) protein, located on chromosome 7. It primarily affects the respiratory and gastrointestinal systems and requires early diagnosis and continuous clinical follow‐up [1, 2].

Chronic pulmonary infections can lead to progressive loss of respiratory function, which represents one of the main causes of mortality. In addition to ventilatory limitation, peripheral muscle weakness associated with nutritional imbalance and systemic inflammation contributes significantly to disease progression [3]. These alterations are associated with exercise intolerance and reduced aerobic fitness, which further contribute to disease worsening [4, 5]. Consequently, this combination of factors may result in poorer physical performance, limitations in activities requiring exertion, and a negative impact on quality of life, and are considered important markers of prognosis and survival [6].

With the development of CFTR modulators, CF has entered a new therapeutic “era.” These drugs act in different ways on specific mutations, promoting improvements in protein function, performance, and stability, thereby limiting disease progression [7, 8]. Pharmacological therapy has brought major advances in the quality of life of people with CF (pwCF) [9], including improved pulmonary function with reduced exacerbation episodes [10], improved nutritional status achieving an ideal body mass index (BMI) [11, 12], and improved exercise tolerance [13]. However, regarding aerobic capacity, some studies have shown improvements in oxygen consumption (VO_2_), whereas others have not reported (NR) significant changes in response to the use of CFTR modulator therapies [7, 14].

Considering the significant clinical and functional changes observed in pwCF in recent years, it is of great importance to describe and analyze the different responses to CFTR modulator therapy, generating relevant information on its potential impact across different domains, including physical and functional capacity. Therefore, the aim of this systematic review and meta‐analysis was to evaluate the effects of CFTR modulator therapy on aerobic fitness in pwCF. These findings have the potential to guide more effective therapeutic and rehabilitation strategies, with the goal of optimizing physical performance and quality of life in this population.

## 2 Methods

This systematic review and meta‐analysis were conducted in accordance with the PRISMA (Preferred Reporting Items for Systematic Reviews and Meta‐Analyses) guidelines. The review protocol was registered in PROSPERO under the number CRD420251066602. No amendments were made to the registered protocol.

### 2.1 Search Strategy

The following databases were searched: Embase, LILACS, PubMed, Scopus, and SciELO. The search terms used were: (“Cystic Fibrosis” OR “mucoviscidosis”) AND (“Elexacaftor‐ivacaftor‐tezacaftor” OR “Trikafta” OR “elexacaftor‐ivacaftor‐tezacaftor drug combination” OR “CFTR modulator” OR “CFTR modulator therapy” OR “cystic fibrosis transmembrane conductance regulator” OR “ETI”) AND (“Exercise Tests” OR “Exercise Testing” OR “Arm Ergometry Test” OR “Cardiopulmonary Exercise Test” OR “Cardiopulmonary Exercise Testing” OR “Bicycle Ergometry Test” OR “Exercise capacity” OR “Aerobic capacity” OR “peak oxygen uptake” OR “VO_2_peak” OR “Cardiopulmonary exercise testing (CPET)” OR “aerobic fitness”). No filters were applied. A manual search of the reference lists of the selected articles was also conducted to identify additional relevant publications. Searches were performed in October 2025.

### 2.2 Study Selection

Two authors (I.S.A. and M.S.C.) independently assessed titles and abstracts to identify eligible studies. Eligibility criteria were defined according to the PICO framework. The population (P) comprised individuals of any age or sex with a genetic diagnosis of CF receiving regular CFTR modulator therapy. The intervention (I) was the CFTR modulator therapy. The comparator (C) consisted of the same individuals before initiation of CFTR modulator therapy. The outcome (O) was aerobic fitness assessed by cardiopulmonary exercise testing (CPET), with peak oxygen uptake (peak VO_2_) as the primary outcome. Eligible study designs included analytical, observational, and experimental studies, such as clinical trials, quasi‐experimental studies, and case series. No restrictions were imposed regarding publication date or language. Exclusion criteria included review articles, conference abstracts, letters to the editor, case reports, studies involving individuals who had undergone lung transplantation, and interventions involving formal exercise programs or the addition of medications beyond CFTR modulators or routine CF treatment. Disagreements regarding study eligibility were resolved by consensus and, when necessary, a third author (M.V.F.D.) was consulted.

### 2.3 Data Extraction

Data extraction was performed independently by two reviewers using a standardized data collection form. Disagreements were resolved by consensus. From each included study, the following data were extracted: first author's name, country, year of publication, sample size, age, type of modulator therapy, duration of modulator therapy use, and CPET variables before and after CFTR modulator therapy.

The primary variable analyzed was peak VO_2_, measured in mL·kg^−1^·min^−1^ or mL·min^−1^. Secondary variables included peak VO_2_ as a percentage of predicted, VO_2_ at the ventilatory threshold (VT_1_), breathing reserve (BR), workload (W), forced expiratory volume in one second (FEV_1_), assessed by spirometry, and BMI, assessed by anthropometry, before and after CFTR modulator therapy. When data were NR, this was indicated as NR. No additional assumptions were made regarding missing information.

### 2.4 Quality Assessment

The quality of the studies was independently assessed by two authors (I.S.A. and M.S.C.) using the Joanna Briggs Institute (JBI) instrument. Additional information was requested from the study authors when required data were unclear or not available in the published reports. The instrument provides a qualitative assessment based on questions that classify each article using the responses “yes,” “no,” “unclear,” or “not applicable.” In addition, the overall certainty of the evidence for each outcome was evaluated using the Grading of Recommendations Assessment, Development, and Evaluation (GRADE) approach.

### 2.5 Data Synthesis and Statistical Analysis

Studies were included in each meta‐analysis if they met the eligibility criteria and reported sufficient quantitative data (sample size, mean, and standard deviation) for the outcome of interest before and after CFTR modulator therapy. Meta‐analyses were conducted separately for each outcome variable. When individual patient data were reported, means and standard deviations were calculated to allow inclusion in the meta‐analysis. When the standard deviation of the change score was NR, it was calculated according to the Cochrane Handbook using an imputed correlation coefficient (r). Sensitivity analyses were performed using correlation coefficients (*r*) of 0.25, 0.50, and 0.75. As the pooled effect estimates were materially unchanged across these assumptions, an *r* value of 0.50 was used in the primary analyses.

For data synthesis, Review Manager software version 5.2 (Cochrane Collaboration, Oxford, UK) was used. The pooled effect measure was the mean difference (MD) in change from baseline to follow‐up. A random‐effects model was applied to compute the pooled MD and its 95% confidence interval. This model was chosen due to the expected clinical and methodological heterogeneity among the included studies. A subgroup analysis was performed including only studies evaluating elexacaftor/tezacaftor/ivacaftor (ETI, Trikafta) to explore whether the pooled effects differed from those observed in the overall analyses. Heterogeneity was assessed using the *I* ^2^ statistic and interpreted according to the guidance of the Cochrane Handbook for Systematic Reviews of Interventions. An *I* ^2^ value of 0%–40% might not be important, 30%–60% may represent moderate heterogeneity, 50%–90% may represent substantial heterogeneity, and 75%–100% may represent considerable heterogeneity. A leave‐one‐out influence analysis was performed by repeating each meta‐analysis while sequentially removing one study at a time to evaluate its influence on the pooled effect estimate and heterogeneity. This procedure followed the recommendations of the Cochrane Handbook for investigating highly influential studies. Results were presented in structured tables and visually displayed using forest plots generated in Review Manager.

Reporting bias (e.g., publication bias) was not formally assessed. Although one outcome included 10 studies, graphical or statistical methods such as funnel plot asymmetry or Egger's regression test were not performed due to methodological considerations related to the pre−post study designs and limitations of the analytical approach implemented in the software.

## 3 Results

### 3.1 Study Selection

**Figure 1:**
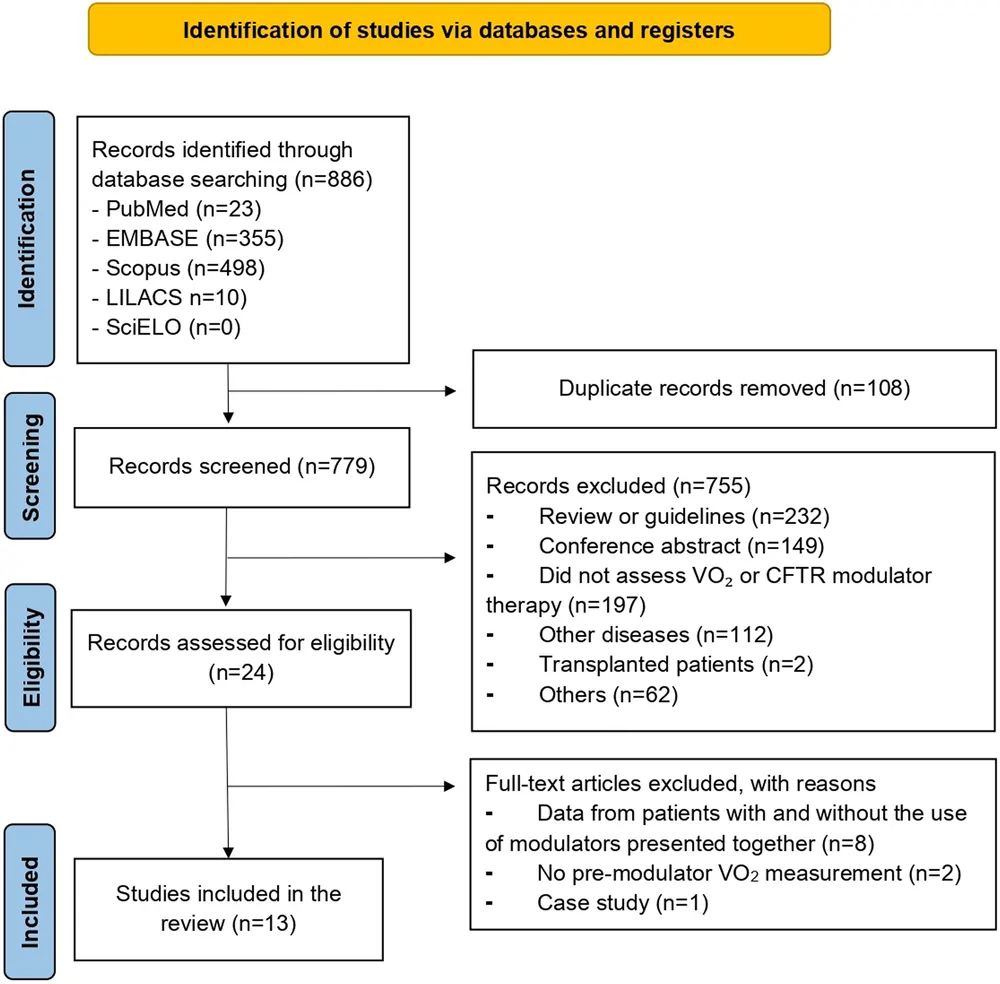
Study selection flow diagram. [Color figure can be viewed at wileyonlinelibrary.com]

A total of 886 articles were identified across the databases. Of these, 108 duplicate records were excluded, and an additional 755 articles were excluded for not meeting the eligibility criteria. The full texts of 14 studies were assessed for eligibility, of which one was excluded for being a case study. Therefore, 13 studies [13, 14, 15, 16, 17, 18, 19, 20, 21, 22, 23, 24, 25] were included in this review, of which 11 studies [13, 14, 15, 16, 17, 20, 21, 22, 23, 24, 25] were included in the meta‐analysis. Figure 1 presents the complete study selection flow diagram.

### 3.2 Characteristics of Protocols and Variables

The selected articles included a total of 578 participants, with sample sizes ranging from 3 to 229 and a mean age ranging from 9.1 to 42.0 years. Of the 13 included studies [13, 14, 15, 16, 17, 18, 19, 20, 21, 22, 23, 24, 25], eight studies [13, 14, 20, 21, 22, 23, 24, 25] evaluated the effects of triple therapy. The duration of modulator use, in weeks, ranged from 6 to 208 weeks. These data are presented in Table 1.

**Table 1:** Main methodological characteristics of the studies included.

Study	Study type	Sample size total/female	Mean age (years)	CFTR Modulator	Time on modulator (weeks)
Savi et al. 2019	Case series	3/0	42.0 ± 15.9*	Lum/Iva	104
Ejiofor et al. 2020	Prospective cohort	21/9	33.0 (23, 40)^$^	Lum/Iva	26
Burghard et al. 2020	Prospective cohort	7/3	15.4 ± 5.8	Iva	52
Wilson et al. 2020	Randomized controlled trial	34/13	24.9 ± 10.2	Lum/Iva	24
Rysgaard et al. 2022	Prospective observational cohort	91/39	23.8 (18.3, 32.3)^$^	Lum/Iva Tez/Iva	26 and 52
Causer et al. 2022	Case series	3/1	14.0 ± 1.5*	Elex/Tez/Iva	6
Gur et al. 2022	Prospective observational cohort	7/3	18.6 ± 4.7	Elex/Tez/Iva	13
Ahmed et al. 2023	Prospective observational cohort	19/13	15.2 ± 2.1	Tez/Iva Elex/Tez/Iva	26 or 35
Philipsen et al. 2024	Prospective observational cohort	229/119	27.0 (17)^$^	Elex/Tez/Iva	52
Stastna et al. 2024	Prospective observational pilot cohort	10/2	14.1 ± 2.8	Elex/Tez/Iva	52
Pérez Ruiz et al. 2025	Prospective observational cohort	28/10	9.1 ± 1.6	Elex/Tez/Iva	26 or 35
Gaupmann et al. 2025	Retrospective observational cohort	25/NR	16.5 ± 2.1	Elex/Tez/Iva	156 or 208
Retucci et al. 2025	Prospective observational cohort	101/39	28.4 ± 8.7	Elex/Tez/Iva	26

*Note:* Data expressed as mean (standard deviation), except for ^$^values are expressed as median (interquartile range or intervals). *Mean and standard deviations calculated by the authors.

Abbreviations: CFTR, cystic fibrosis transmembrane conductance regulator; elex, elexacaftor; iva, ivacaftor; lum, lumacaftor; tez, tezacaftor.

Table 2 presents the results of the main variables analysed in this study. Among the 13 included articles, seven studies [15, 16, 19, 20, 21, 24, 25] assessed peak VO_2_ expressed in mL·min^−1^, of which six studies [15, 16, 19, 21, 24, 25] reported an increase in values. Regarding peak VO_2_ measured in mL·kg^−1^·min^−1^, only one study [15] did not report this variable, resulting in six studies [13, 15, 19, 21, 22, 24] that documented improvements in this outcome. peak VO_2_ as a percentage of predicted was reported in seven studies [13, 15, 17, 20, 21, 22, 25], of which five studies [13, 15, 20, 21, 22] demonstrated an increase. VO_2_ at VT_1_ was assessed in nine articles [13, 14, 15, 18, 20, 22, 23, 24, 25], with improvements observed in five of them [14, 15, 18, 20, 22]. Among the 13 included articles, seven studies [13, 14, 15, 20, 23, 24, 25] analysed BR, showing increased results in four studies [14, 20, 23, 25]. Workload was assessed in 10 articles [13, 14, 15, 16, 17, 19, 21, 22, 24, 25], with improvements reported in eight of them [13, 16, 17, 19, 21, 22, 24, 25]. Finally, FEV_1_ was reported in all 13 included studies, with increased values described in 10 studies [13, 14, 15, 17, 19, 20, 21, 23, 24, 25].

**Table 2:** Main results of the variables included in the systematic review.

Study		BMI (kg.m^2^)	FEV_1_ (%)	VO_2_ (mL.min^−1^)	VO_2_ (mL.kg^−1^.min^−1^)	VO_2_ (% pred)	VO_2_ VT_1_ (%)	BR (%)	Workload (W)
Savi et al. 2019	Pre* Post* MD*	NR NR 5.3 ± 4.9^&^	NR NR 0.7 ± 8.3^&^	1631.7 ± 232.5 1980.0 ± 410.0 348.3 ± 471.4	24.9 ± 4.9 29.0 ± 7.0 4.1 ± 8.5	60.8 ± 5.7 79.7 ± 7.6 18.9 ± 9.5	NR NR 25.3 ± 12.8^&^	13.6 ± 11.8 −3.9 ± 30.9 −17.5 ± 33.1	160.0 ± 30.0 160.0 ± 30.0 0.0 ± 0.0
Ejiofor et al. 2020	Pre Post MD	−1.7 (−2.5, −0.5)^#$^ NR NR	38.7 (32.2, 57.6)^$^ NR NR	1408.6 ± 296.0 1497.6 ± 266.4 89 (−33.1, 211.1)^$$^	NR NR NR	NR NR NR	NR NR NR	NR NR NR	135.7 ± 42.6 148.0 ± 48.1 12.3 (0.6, 24.0)^$$^
Burghard et al. 2020	Pre Post MD	19.9 ± 3.4 21.2 ± 3.4 NR	81.7 ± 17.8 97.7 ± 19.6 NR	2300.0 ± 1000.0*** 2100.0 ± 800.0*** NR	42.9 ± 6.6 36.3 ± 5.6 NR	93.4 ± 16.5 80.7 ± 12.0 NR	NR NR NR	NR NR NR	174.9 ± 83.5 191.3 ± 86.2 NR
Wilson et al. 2020	Pre Post MD	21.1 ± 3.0 NR 0.5 ± 0.2	65.6 ± 15.0 NR −0.6 ± 1.7	NR NR NR	31.8 ± 8.8 NR −2.7 ± 0.7	NR NR NR	1147.7 ± 514.3** NR 1.8 ± 4.8	NR NR NR	NR NR NR
Rysgaard et al. 2022	Pre^$$^ Post MD^$$^	NR NR NR	75.9 (70.9, 80.9) NR 3.5 (1.8, 5.3)	1940.0 (1825, 2055) NR 145.7 (91.2, 200.2)	32.6 (31.0, 34.2) NR 1.07 (0.2, 2.0)	NR NR NR	NR NR NR	NR NR NR	180.1 (168.4, 191.8) NR 14.2 (9.1, 19.2)
Causer et al. 2022	Pre* Post* MD*^&^	18.5 ± 1.8 21.2 ± 1.7 2.7 ± 2.5	62.5 ± 17.1 76.3 ± 19.8 13.8 ± 26.2	1800.0 ± 600.0*** 2400.0 ± 400.0*** 600.0 ± 700.0***	37.5 ± 14.1 42.9 ± 14.3 5.4 ± 20.1	74.0 ± 25.5 95.7 ± 29.0 21.7 ± 38.6	61.3 ± 18.3 54.0 ± 13.8 −7.3 ± 22.9	18.1 ± 4.9 14.7 ± 10.8 −3.4 ± 11.9	116.0 ± 38.4 125.0 ± 38.0 9.0 ± 54.1
Gur et al. 2022	Pre Post MD	19.4 ± 2.6 20.3 ± 2.2 NR	58.4 ± 26.9 70.2 ± 27.1 NR	1864.4 ± 603.0 1861.4 ± 501.2 NR	32.6 ± 9.0 32.4 ± 7.1 NR	76.7 ± 19.9 78.4 ± 16.3 NR	38.6 ± 9.3 41.3 ± 13.0 NR	21.7 ± 11.0 28.6 ± 14.3 NR	NR NR NR
Ahmed et al. 2023	Pre Post MD	19.9 ± 3.2 20.7 ± 3.5 NR	86.0 ± 12.3 94.7 ± 11.3 NR	NR NR NR	33.1 ± 6.7 30.4 ± 7.9 NR	NR NR NR	47.6 ± 15.4 52.9 ± 12.7 NR	30.3 ± 12.3 43.7 ± 14.4 NR	75.2 ± 13.4 72.7 ± 13.5 NR
Philipsen et al. 2024	Pre Post MD^$$^	21.7 ± 3.8 23.1 ± 3.8 1.4 (1.1, 1.6)	75.9 ± 22.8 87.8 ± 22.1 11.9 (10.4, 13.4)	1969.3 ± 588.4 2124.2 ± 635.6 154.9 (118.9, 190.9)	31.9 ± 8.3 32.4 ± 8.6 0.6 (0.1, 1.1)	88.6 ± 20.0 92.3 ± 20.2 3.7 (2.3, 5.2)	NR NR NR	NR NR NR	174.8 ± 60.4 187.8 ± 88.9 13.0 (3.9, 22.1)
Stastna et al. 2024	Pre Post MD	NR NR NR	89.4 ± 10.9 NR NR	1900.0 ± 800.0*** 2700.0 ± 700.0*** 800.0 ± 600.0***	34.8 ± 7.7 40.5 ± 5.7 5.7 ± 8.1	78.5 ± 18.5 94.4 ± 12.0 15.9 ± 16.6	45.9 ± 0.2 60.9 ± 0.1 15.0 ± 0.1	NR NR NR	178.4 ± 64.8 202.6 ± 54.1 24.2 ± 36.1
Pérez Ruiz et al. 2025	Pre Post MD	−0.4 ± 0.9 −0.3 ± 0.7^#^ NR	−0.0 ± 1.0 0.9 ± 1.5^#^ NR	NR NR NR	38.6 ± 7.8 36.9 ± 8.6 NR	NR NR NR	22.6 ± 4.4 20.1 ± 3.6 NR	17.5 ± 18.6 33.4 ± 20.8 NR	NR NR NR
Gaupmann et al. 2025	Pre^#^ Post^##^ MD	0.0 ± 0.7^#^ 0.3 ± 0.9& NR	−1.2 ± 1.6^#^ −0.8 ± 1.3& NR	1692.0 ± 387.0 2201.0 ± 710 NR	34.0 ± 6.0 36.0 ± 7.0 NR	NR NR NR	26.4 ± 2.5 24.6 ± 2.2 NR	44.0 ± NR 23.0 ± NR NR	122.0 ± 38.0 176.0 ± 60 NR
Retucci et al. 2025	Pre Post MD	−0.2 ± 0.9 ^#^ NR NR	75.2 ± 23.1 89.2 ± 22.9 NR	1739.4 ± 497.5 1761.2 ± 524.3 NR	28.6 ± 6.4 28.0 ± 6.8 NR	73.2 ± 17.0 72.8 ± 16.7 NR	57.4 ± 15.2* 55.5 ± 16.2* NR	34.2 ± 22.63 9.0 ± 17.5 NR	151.0 ± 38.6 156.6 ± 41.1 NR

*Note:* BMI, body mass index; BR, breathing reserve; FEV_1_, forced expiratory volume in the first second; NR, not reported; VO_2_, oxygen consumption; VT_1_, ventilatory threshold; W, watt.

^&^Values presented in *z*‐score; ^&^Data presented as percent change.

*Mean and standard deviations calculated by the authors. Data expressed as mean (standard deviation), except for ^$^values are expressed as median (interquartile range or intervals). ^$$^Values are expressed as mean (confidence interval).

**Values expressed in mL.min^−1^. ^&^Data corresponding to the period 0–1 year before the modulator.

^&&^Data corresponding to years 1 and 2 after the modulator. ***Values originally reported in L.min^−1^ were converted to mL.min^−1^ by the authors.

### 3.3 Meta‐Analyses

**Figure 2:**
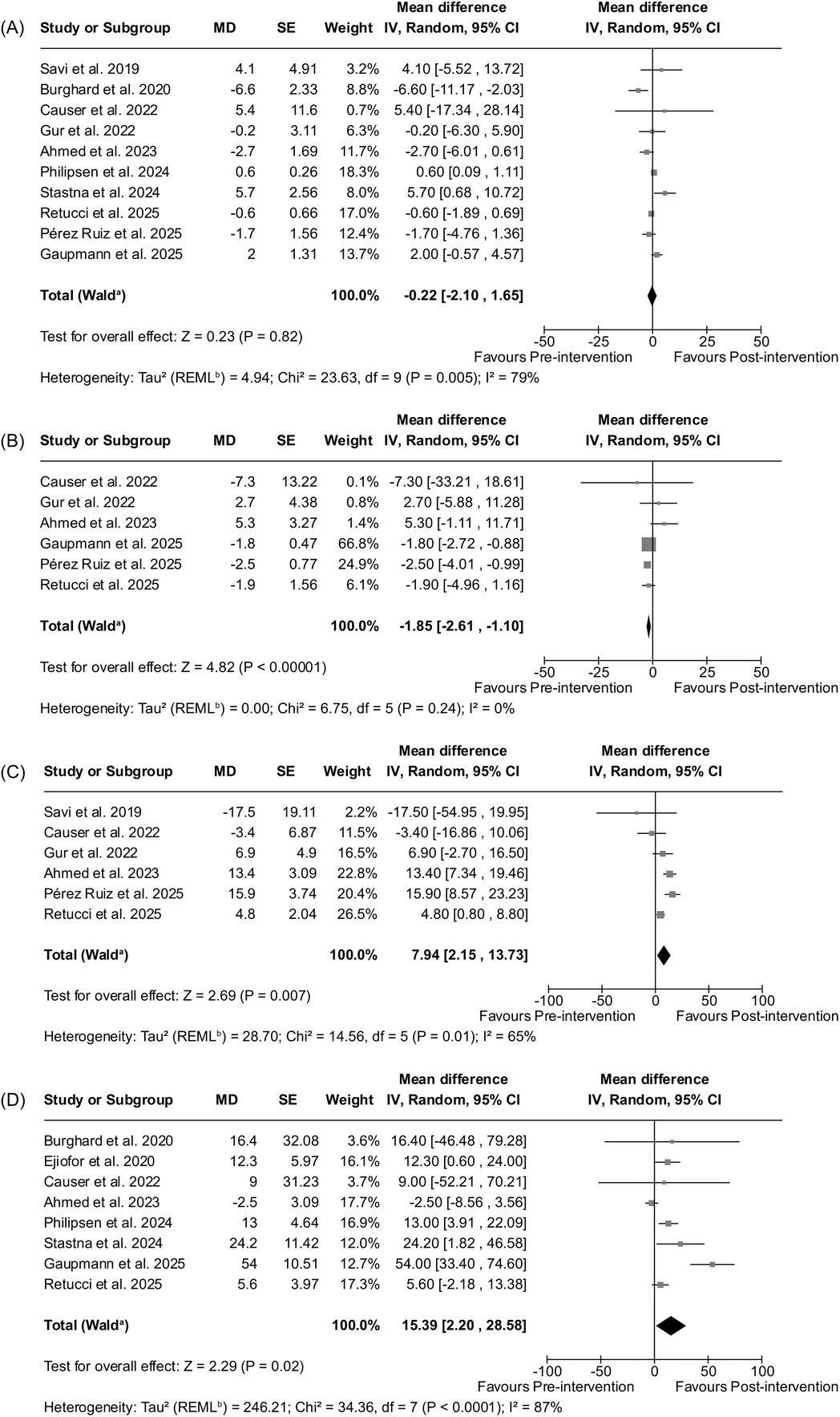
Forest plots of mean differences (random‐effects model) between pre and post CFTR modulator values for the following outcomes: (A) peak VO_2_ (mL·kg^−1^·min^−1^); (B) VO_2_ at the first ventilatory threshold (VT_1_, % of peak VO_2_); (C) breathing reserve (BR, %); and (D) maximal workload (W).

The quantitative syntheses included predominantly observational studies with small to moderate sample sizes. The risk of bias assessment is described in the Supporting Information Materials. The meta‐analysis included 10 studies for the primary outcome, assessing differences in peak VO_2_ (mL·kg^−1^·min^−1^) between the pre and post‐ CFTR modulator periods. Figure 2A presents the analysis of peak VO_2_ (mL·kg^−1^·min^−1^), in which no significant difference was observed between the pre‐ and post‐intervention periods (MD = −0.22; 95% CI: −2.10 to 1.65; *p* = 0.82), with a heterogeneity of *I* ^2^ = 79% (*p* = 0.005). When analysing VO_2_ at VT_1_ (Supporting Information Materials), no significant difference was observed between the pre‐ and post‐intervention periods (MD = 2.40; 95% CI: −3.26 to 8.06; *p* = 0.41). However, considerable and statistically significant heterogeneity was observed among the included studies (*I* ^2^ = 99%; *p* < 0.00001). Based on these findings, a sensitivity analysis was performed, which identified a specific study with a much larger effect than the others, supporting its exclusion from the final analysis. Thus, Figure 2B presents the final meta‐analysis after sensitivity analysis, demonstrating a significant difference in VO_2_ at VT_1_ between the pre‐ and post‐intervention periods (MD = −1.85; 95% CI: −2.61 to −1.10; *p* < 0.00001), favouring the pre‐intervention period. No heterogeneity was observed among the included studies (*I* ^2^ = 0%; *p* = 0.24), indicating high consistency of the results after exclusion of the study responsible for the heterogeneity. Regarding BR, presented in Figure 2C, a significant difference was observed after CFTR modulator therapy (MD = 7.94; 95% CI: 2.15 to 13.73; *p* = 0.007), although with considerable heterogeneity among studies (I^2^ = 65%; *p* = 0.01). When workload was analysed (Figure 2D), a significant increase after CFTR modulator therapy was observed (MD = 15.39; 95% CI: 2.20 to 28.58; *p* = 0.02). The heterogeneity analysis demonstrated considerable variability among the included studies (*I* ^2^ = 87%; *p* < 0.0001). The subgroup analysis (Supporting Information Materials), restricted to studies evaluating ETI, showed results consistent with those of the overall analysis.

### 3.4 Influence Analysis

The influence analysis identified that the removal of the study by Stastna et al. [22] reduced heterogeneity from *I* ^2^ = 99% to *I* ^2^ = 0%, indicating a strong impact of this study on the variability of results for the VO_2_ at VT_1_ outcome.

### 3.5 Methodological Quality

Regarding the methodological quality of the included studies, the two case series studies [13, 15] received between seven and eight questions rated as “yes” and two as “unclear.” The cohort studies [14, 16, 17, 19, 20, 21, 22, 23, 24, 25] received between five and eight questions rated as “yes,” between one and four questions rated as “no,” and three rated as “unclear.” The randomized controlled trial [17] received “yes” for all questions. Overall, the JBI assessment indicated that most studies met the majority of the methodological quality criteria. The randomized controlled trial met all quality criteria, whereas the case series and cohort studies showed some methodological limitations, mainly related to participant selection, management of confounding factors, and completeness of follow‐up. These limitations should be considered when interpreting the findings, although appropriate statistical analyses were performed and valid outcome measurements were consistently reported across most studies. The complete results, including tables with the assessed questions, are described in the Supporting Information Materials.

According to the GRADE approach, the certainty of the evidence for the evaluated outcomes ranged from low to very low. For the primary outcome peak VO_2_ (*n* = 10 studies, 432 participants), the evidence was graded as very low due to inconsistency and imprecision, showing no significant difference compared to baseline. Similarly, very low certainty evidence was observed for BR (*n* = 6 studies) and workload (*n* = 8 studies), while VO_2_ at VT_1_ (*n* = 6 studies) presented low certainty evidence.

For the specific subgroup of participants treated with ETI, the certainty of evidence also ranged from low to very low. The analysis for peak VO_2_ (*n* = 7 studies, 403 participants) indicated very low certainty evidence due to imprecision, showing an increase that was not statistically significant compared to baseline. Similarly, very low certainty evidence was found for breathing reserve (*n* = 4 studies) and workload (*n* = 5 studies), while VO_2_ at VT_1_ (*n* = 5 studies) showed low certainty evidence. The complete results are described in the Supporting Information Materials.

## 4 Discussion

This systematic review and meta‐analysis aimed to evaluate the effects of CFTR modulator therapy on aerobic fitness in pwCF. Overall, the results indicated that there was no consistent improvement in peak VO_2_ after initiation of modulator therapy, whereas some submaximal outcomes related to ventilatory response and exercise performance showed variable changes. To the best of our knowledge, this is the first systematic review and meta‐analysis to specifically investigate the effects of CFTR modulators on aerobic fitness and variables derived from CPET, thereby filling a gap in the literature and contributing to a more comprehensive understanding of the functional impacts of this therapy.

The introduction of CFTR modulators represented a milestone in the treatment of CF, with consistent benefits in relevant clinical outcomes, including improved pulmonary function, reduced exacerbations, and improvements in nutritional status and quality of life, as described in systematic reviews and meta‐analyses and supported by previous clinical trials [26, 27]. Despite these well‐established benefits, the impact of this therapy on aerobic fitness remains poorly investigated. Studies that specifically evaluated peak VO_2_ expressed in mL·kg^−1^·min^−1^ after CFTR modulator use have shown inconsistent results, with some observational studies reporting increases in this variable [13, 15, 19, 21, 22, 24], whereas other studies, including those with larger samples and more robust designs, did not confirm this finding [14, 17, 18, 20, 23, 25]. The meta‐analysis of these studies demonstrated that the use of CFTR modulators did not promote a significant improvement in peak VO2 when comparing the pre‐ and post‐intervention periods. This finding may be related to the multifactorial nature of peak VO_2_, which depends not only on pulmonary function but also on central and peripheral adaptations to exercise, including muscle oxidative capacity, cardiovascular efficiency, and overall physical conditioning [3]. Previous studies in pwCF have shown that improvements in pulmonary function do not always translate into proportional increases in peak VO_2_, particularly in the absence of interventions that stimulate peripheral adaptations, such as structured physical training [28]. Therefore, although CFTR modulators provide clinically relevant benefits, their isolated action may be insufficient to induce consistent gains in maximal aerobic fitness [21, 28].

Regarding secondary outcomes, previous evidence indicates that variables such as the first ventilatory threshold, BR, and workload have high clinical relevance in CF, as they often reflect functional limitations not captured by peak VO_2_ [29, 30]. Prior studies have shown that pwCF exhibit early ventilatory thresholds and ventilatory inefficiency during exercise, which are associated with disease severity, even in the presence of preserved peak VO_2_ values [29]. VT_1_ has been described as a sensitive marker of submaximal aerobic fitness and peripheral adaptations, with direct implications for exercise training prescription and monitoring in this population [29, 31]. In the present meta‐analysis, VO_2_ at VT_1_ was significantly lower after CFTR modulator therapy following sensitivity analysis. Although this difference reached statistical significance, its magnitude was small, and its clinical relevance remains uncertain. Therefore, this finding should be interpreted with caution. BR is widely used to identify ventilatory limitation during exercise and is related to pulmonary mechanics and the severity of respiratory dysfunction [31]. In the present meta‐analysis, BR increased significantly after CFTR modulator therapy, whereas no significant improvement was observed in peak VO_2_. This apparent dissociation may indicate that improvements in ventilatory function alone are not sufficient to produce measurable gains in maximal aerobic fitness. Although the mechanisms underlying this apparent dissociation remain unclear, the present findings support this hypothesis and warrant further investigation. Workload reflects overall functional capacity and peripheral adaptations, being associated with performance in activities of daily living and sensitive to interventions even in the absence of proportional changes in peak VO_2_ [18, 32, 33, 34]. In the present meta‐analysis, workload increased significantly after CFTR modulator therapy. However, considerable heterogeneity was observed among the included studies, and this finding should therefore be interpreted with caution. Consistent with this physiological basis, the findings of this review reinforce the importance of assessing submaximal and functional parameters, in addition to peak VO_2_, to achieve a more comprehensive understanding of responses to CFTR modulator therapy.

Several factors may have influenced the results of this review, including the duration of CFTR modulator use, which ranged from 6 to 208 weeks among the included studies. Peak VO_2_ is an outcome that reflects complex physiological adaptations and depends on specific peripheral stimuli. Findings from systematic reviews and meta‐analyses in pwCF indicate that consistent increases in peak VO_2_ tend to be observed after approximately 8−12 weeks of interventions, a period required for cardiovascular and muscular adaptations to exercise to occur [28]. Thus, although CFTR modulators may promote improvements in pulmonary clinical status, the duration of pharmacological treatment alone may be insufficient to induce measurable aerobic adaptations, particularly in the absence of exercise‐specific stimuli, which may contribute to the lack of consistent improvement in peak VO_2_ observed in this meta‐analysis [21, 28]. In addition, the included studies evaluated different types of CFTR modulators with distinct mechanisms of action and clinical responses. While more recent modulators, especially ETI, have been associated with greater improvements in clinical outcomes and pulmonary function, their effects on aerobic fitness remain variable. In this review, the primary analyses were conducted by pooling studies regardless of the type of CFTR modulator used, including ivacaftor monotherapy, dual therapies, and triple therapy, which may have contributed to the observed heterogeneity and limited the identification of potential differences among modulator classes [11, 35, 36]. However, the subgroup analysis restricted to studies evaluating ETI yielded findings similar to those of the overall analysis. Methodological heterogeneity was also relevant, including differences in CPET protocols, outcome definitions, and clinical characteristics of the populations evaluated, such as age, disease severity, and physical activity level. Although CF is a clinically heterogeneous condition, this variability persists despite the use of CFTR modulators. The absence of stratified analyses for these variables in the included studies limits the interpretation of the findings and hinders direct comparisons across studies.

Some limitations should be considered when interpreting the results of this review. Most of the included studies had observational designs, small sample sizes, and short follow‐up periods. The inclusion of two case series with only three participants each may have contributed to less precise and potentially unstable effect estimates. In addition, substantial heterogeneity was observed in several outcomes. To address this variability, random‐effects models were used, as recommended by the Cochrane Handbook. Additionally, leave‐one‐out sensitivity analyses were performed, allowing assessment of the individual influence of studies on the pooled results and the robustness of the findings.

In conclusion, the results of this review suggest that treatment with CFTR modulators is not consistently associated with improvements in aerobic fitness, as assessed by peak VO_2_, although improvements were observed in some secondary outcomes related to ventilatory response and exercise performance, including BR and maximal workload. These findings suggest that assessments based exclusively on peak VO_2_ may underestimate clinically relevant functional changes after initiation of CFTR modulator therapy. Future studies with greater methodological standardization, longer follow‐up, and better control of physical training levels are needed to elucidate the long‐term effects of CFTR modulators on aerobic fitness in pwCF.

## Author Contributions

**Ingrid Silveira de Almeida:** conceptualization, investigation, writing – original draft, methodology, formal analysis. **Fernanda Maria Vendrusculo:** conceptualization, methodology, writing – review and editing; supervision. **Mariana Severo da Costa:** investigation, writing – review and editing. **Márcio Vinícius Fagundes Donadio:** conceptualization, investigation, writing – review and editing, methodology, formal analysis, project administration, supervision.

## Conflicts of Interest

The authors declare no conflicts of interest.

## Data Availability

The data collection template, the data extracted from included studies, and any data used for analyses will be made available upon reasonable request from the corresponding author. Any analytic code and other materials used in the review can also be obtained in the same way.
